# The Multifaceted Comparison of Effects of Immobilisation of Waste Imperial Smelting Furnace (ISF) Slag in Calcium Sulfoaluminates (CSA) and a Geopolymer Binder

**DOI:** 10.3390/ma17133163

**Published:** 2024-06-27

**Authors:** Beata Łaźniewska-Piekarczyk, Monika Czop, Jan Antoni Rubin

**Affiliations:** 1 Department of Building Processes and Building Physics, Faculty of Civil Engineering, The Silesian University of Technology, Akademicka 5, 44-100 Gliwice, Poland; 2 Department of Technologies and Installations for Waste Management, Faculty of Energy and Environmental Engineering, The Silesian University of Technology, Konarskiego 18, 44-100 Gliwice, Poland; monika.czop@polsl.pl; 3 Institute of Architecture, State University of Applied Sciences in Racibórz, Akademicka 1, 47-400 Racibórz, Poland; jan.rubin@akademiarac.pl

**Keywords:** ISF slag, CSA cement, geopolymer, immobilisation, leachability

## Abstract

Using waste materials as replacements for sand in building materials helps reduce waste and improve the properties and sustainability of the construction materials. Authors proved the possibility of using imperial smelting furnace (ISF) slag granules as a 100% substitute for natural sand in self-compacting (SCC) cement-based mortars of calcium sulfoaluminates (CSA). The study proved that ISF slag’s radioactive properties meet this area’s requirements. CSA cement eliminates the noted problem in the case of concrete with Portland cement, which is the extended setting of the cement binder. The research findings indicate that using slag to replace sand up to 100% in mortars without grains smaller than 0.125 mm allows high flowability, compaction, low porosity and mechanical parameters. The compressive strength of the CSA cement mortars was about 110 MPa, and more than 140 MPa for geopolymer mortar. Unfortunately, the alkaline pH of a geopolymer causes high leachability of barium and sodium. Thus, the CSA cement is in a more favourable binder to achieve high strength, is environmentally friendly, and is a self-compacting mortar or concrete.

## 1. Introduction

**Figure 1:**
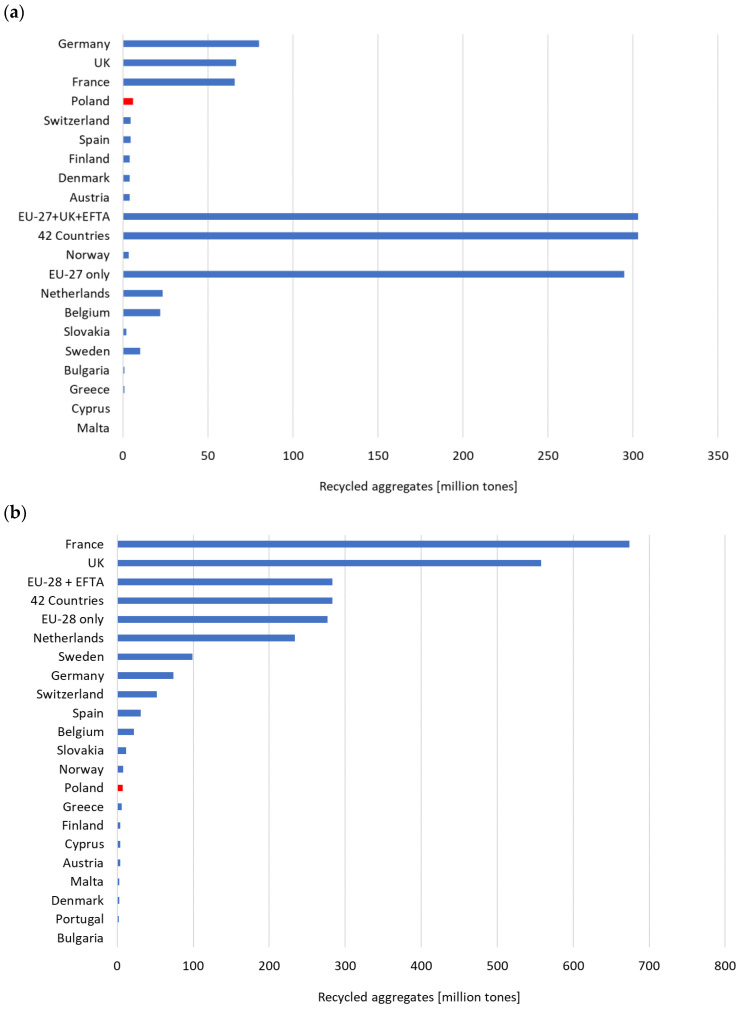
Recycled aggregates in tons (EU27 + UK + EFTA) in (**a**) 2022, (**b**) 2020 (Source: Aggregates Europe UEPG 2022) [6]. Red part = Poland.

Despite its widespread availability, natural sand faces various resource-related issues that may impact its long-term viability as a raw material [1,2,3,4,5]. In developed economies with high ecological awareness, alternative aggregates produce a significant portion of total aggregate production. This is illustrated in Figure 1; the following years brought an increasing use of recycled aggregates in concrete. In 2022 (Figure 1a), countries like Great Britain (with 68 million tons) and the Netherlands (with 18 million tons) produce around 25% of their total aggregate production as alternative aggregates. Other countries like Belgium (16 million tons), Germany (the largest producer with nearly 100 million tons), and Denmark (8 million tons) also have a share of over 15% of total production. However, alternative aggregates produce only about 3.5% of the total aggregate output in Poland. On average, the share of artificial aggregates for EU-27 countries is almost 9% [6].

Using alternative aggregates aligns with waste management’s primary goals and principles [6]. These goals include reducing waste volume and environmental impact and recovering through environmental protection principles [7,8]. Additionally, it aids in protecting natural aggregate deposits. “Alternative aggregates” describe these raw materials’ origins and roles.

Imperial smelting furnace (ISF) slag was identified as a promising material for concrete production [9,10,11]. Research proved that adequate ISF slag can be used as an aggregate or cement production additive to improve its mechanical properties [9,12]. Research results show that ISF slag can be used to improve the resistance of concrete. ISF slag enhances the resistance of cementitious materials to corrosion caused by carbonation. Due to the heterogeneity of the shape of slag particles, the porosity decreases, and the alkalinity of the cement matrix increases, which slows down the diffusion of carbon dioxide into concrete. Nevertheless, from the resistance and mechanical parameters, from a concrete point of view, the proportion of ISF slag to natural aggregate is essential. The potential corrosion rate tests have shown that the best protective properties are found in mixtures where the sand was replaced with ISF slag at up to 10%. The disadvantage is that the mixtures showed reduced abrasion resistance when ISF slag was used as fine aggregate, even at 10% utilization. With this application, mining and environmental problems related to ISF storage can be effectively solved, thus making the zinc industry more sustainable [12]. The best properties protecting against the penetration of chloride ions are demonstrated by concrete made using ISF slag as a sand substitute in amounts up to 25%. The moderate replacement of sand with ISF slag limited to 25% effectively inhibits corrosion processes in these concretes [13,14]. However, their effect on cement binding is challenging to use on a larger scale [9,13,15]. It can be concluded that ISF slag is a safe replacement for 50% of fine aggregate in producing durable concrete with sufficient mechanical properties. This type of concrete is resistant to corrosion and acids when the slag content is up to 50%. Based on a comparison of research results presented by the publication [16], ISF slag can also replace around 50% of conventionally used fine aggregate in concrete production. Such concrete is also resistant to corrosion and acid. However, the durability parameters of the concrete mixes are reduced with 60% utilisation of ISF. The abrasion resistance of the slag-concrete mixtures decreases slightly when ISF slag is used in amounts up to 50%. The water absorption of the slag-concrete mixtures decreases as sand is replaced with slag. However, the porosity of the concrete mixtures remains mostly the same after sand is replaced with ISF slag. With 60% use of ISF, the strength parameters of concrete mixtures with Portland cement are reduced. However, properties like flexural strength and pull-off strength remain within acceptable limits. Due to the rough texture of ISF particles, the interfacial transitional zone (ITZ) formed between ISF slag and cement particles is denser, reducing water absorption by 20–25% in mixes with 60% ISF slag. Moreover, heavy metals in ISF slag are effectively bound in the cement matrix, preventing heavy metal contamination of the environment [15]. Nonetheless, mixes showed reduced resistance to abrasion when ISF slag is used as fine aggregate, even at 10% utilisation.

Unfortunately, as was proved in the cited above research, the addition of waste slag, like ISF slag, which contains heavy metals [17,18,19,20], significantly delays the setting of mortars and concretes with Portland cement [13,15], which can be avoided if the slag particles are coarser than 0.3 mm. This is the most critical, unsolved problem in producing mortar or concrete with a large volume of ISF slag. The authors propose a new proposition for using ISF slag as an aggregate. Instead of Portland cement, the quick-setting cement, aluminium sulphate cement (CSA), is proposed. The use of CSA cement in producing mortar or concrete allows for short setting time (<30 min with natural aggregate) and very high early strength, with up to 25 MPa achieved after just 6.5 h of solidification. Composites made using CSA cement are highly durable, with frost resistance comparable to those made with Portland cement. They also exhibit low chloride permeability and limited carbonation. CSA concrete also offers high freeze resistance, exceeding 200 cycles, due to its tightness, preventing permeability under a pressure of 30 kg/cm^2^. This makes CSA concrete resistant to contact with magnesium and sodium chloride salts. CSA concrete is not resistant to acids and high temperatures exceeding 150 °C.

The mortars were designed to be self-compacting, so they did not need mechanical compaction to reduce production costs and CO_2_ emissions associated with SCC. The authors designed the cement and geopolymer mortars up to the content of 100% replacement of sand by ISF slag, but before use, the slag was adequately prepared. The mortars were compared due to flowability, the waste aggregate test’s radioactivity, the cement’s porosity and a geopolymer self-compacting mortars, and mechanical properties were investigated. 

Nevertheless, the key objective of the research program was to estimate the impact of the cement or geopolymer mortars with ISF slag on the natural environment. The research analysis focuses on the effect of slag on the strength of self-compacting cement mortars with CSA cement and geopolymer self-compacting mortars with increased stability and their impact on the natural environment. The research results [17,21,22,23,24] also suggest that geopolymerization is beneficial for utilizing ISF slag. The authors critically verified the possibility of using ISF slag in the geopolymer matrix. 

## 2. Materials and Methods

### 2.1. Properties of ISF Slag

ISF slag has various potential applications as concrete aggregate, but unfortunately, the effects depend on its chemical composition and physical properties. Analyzed ISF slag comes from Polish Zinc Smelter (Świętochłowice, Poland), which annually produces around 30 thousand tons of ISF slag. However, introducing ISF slag into concrete can be a valuable way to recycle and reduce waste, but this must be conducted with care to prevent the introduction of any harmful ingredients that could potentially harm the quality of the mortar or concrete, as well as the environment [3,12,25].

ISF slag is waste material with code 10 05 01—waste from zinc metallurgy. 10 05 01. Slags from primary production and secondary (except 10 05 80) 17 01 06 are considered heterogeneous substances [11,26,27]. Imperial smelting furnace (ISF) slag is one among many other materials that, when dumped as such, can cause severe environmental damage due to the presence of heavy metals in it [9,15,16,17,18,19,20,21,22,23,24,25,26,27,28]. 

In the first stage of the research, the chemical composition of the slag and its natural radioactivity acc. the ITB Instruction guidelines [29] were investigated. Raw materials used for feeding, such as Zn-Pb concentrates, contain several accompanying elements besides the main ones, such as Fe, Cu, Cd, Hg, As, Sb, Bi, and Tl [26,30,31,32,33]. These concentrates are produced from recast furnaces, fluxes, and returnable materials. Zinc is separated from lead and slag by melting the charge at temperatures between 1300 °C and 1350 °C, known as the reduction and distillation process. The hot slag is then immediately water granulated. However, the speciation of zinc, lead, copper, and arsenic in the slag controls its recovery or fate in the environment, and this has not been thoroughly investigated in the literature. X-ray Absorption Spectroscopy (XAS) was used for the first time on this complex, poorly crystalline material to better understand the speciation of elements at low concentrations. Zn, Cu, As K-edge and Pb L3-edge XAS were carried out for a Pb/Zn slag from a closed ISF facility in England, supported by Fe, S, and P K-edge XAS. The results are presented in the context of a complete literature review. X-ray fluorescence showed that concentrations of Zn, Pb, Cu, and As were 8.4%, 1.6%, 0.48%, and 0.45%, respectively. XAS provided a complete understanding of the matrix, although Wüstite (FeO) was the only crystalline phase identified by X-ray diffraction. Zn was found to be mainly present in glass, ZnS, and possibly solid solutions with Fe oxides; Pb was primarily present in glass and apatite minerals (e.g., Pb_5_(PO_4_)_3_OH); Cu was mainly speciated as Cu_2_S, with some metallic Cu and a weathering product, Cu(OH)_2_; As speciation was likely dominated by arsenic (III) and (V) oxides and sulphides. 

**Figure 2:**
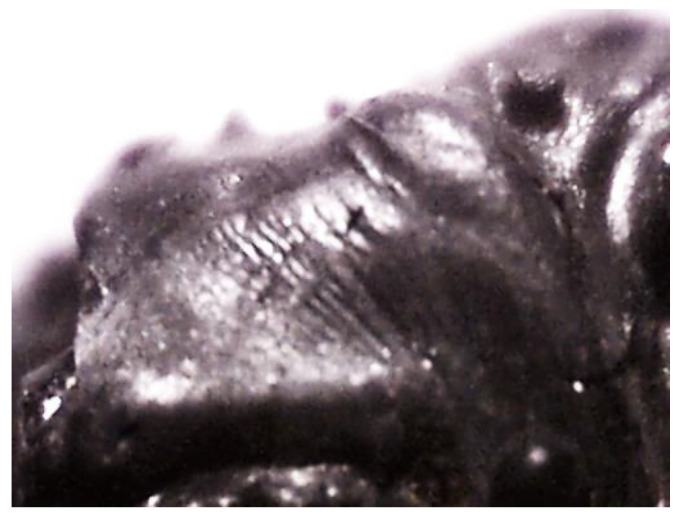
Microscopic photo (60) of the surface of a single grain of ISF slag; scale: 60 times magnified view.

**Figure 3:**
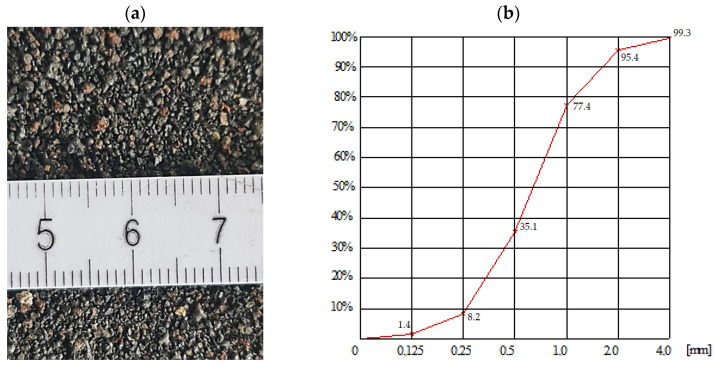
ISF slag, (**a**) view of slag grains with diameters 0.125–2.0 mm, (**b**) ISF slag granulometric curve of mortars.

Fortunately, the granulated ISF slag contains various chemical and mineral components that are melted and consolidated to form a glaze (Figure 2). This glaze binds and scatters heavy metals, primarily zinc and lead, in the slag (as depicted in Figure 3). On the other hand, iron is chemically bound to calcium in the form of amorphous iron-calcium silicates. Due to its chemical composition, the ISF slag granulate has a bulk density of 3.8 kg/dm^3^. Based on the analyses performed, the chemical composition of the slag and the share of individual compounds were determined (Table 1). Table 2 contains the results of determining the pH of the slag.

As research results in Table 3 indicated, the aluminium content percentage of Al_2_O_3_, MgO, and SO_3_ does not meet the guidelines set at 12% or less. Based on the calculated value of the modulus M = 4.50, the tested slag can be classified as an aggregate of the silicon-aluminium-ferrous variety. Table 3 lists the compounds’ content leached from the EC Zabrze and ISF slag.

The granulometric composition of the slag using the PN-EN 933-1:2012 method [35]. The ISF slag was used as a 100% substitute for natural sand in self-compacting cement mortar and a geopolymer, but before use, the slag was thoroughly rinsed and then dried. The minor gains (smaller than 0.125 mm) of the ISF slag were eliminated to obtain high-strength mortar. Figure 3 provides a view of the slag grains and the granulometric composition acc. to EN 933-1:2012 [36].

That is important also, as per construction standards, all aggregates utilized for concrete and earthworks must conform to the guidelines specified in ITB Instruction № 234 [29] regarding the concentration of radioactive elements. The presence of natural radioactive isotopes, such as potassium K-40, radium Ra-226, and thorium Th-228, in the raw materials and building materials intended for human or animal habitation, as well as in industrial waste used for construction, are assessed using two activity indexes. These indexes are (a) activity index f_1_, which measures the content of natural radioactive isotopes, and (b) activity index f_2_, which measures the content of radium Ra-226. According to Polish legal regulations [30], which comply with the rules of the European Union (Regulation of the Council of Ministers of 2 January 2007) [35,36], the activity indicators are subject to two limitations: the values of activity indicators f_1_ and f_2_ cannot exceed the value more than 20% [30,35].

The following information pertains to the levels of radioactivity in various types of materials used in construction for human or livestock habitation, as well as levelling of areas for development [37]:

- For raw and construction materials, f_1_ = 1.0 and f_2_ = 200 Bq/kg.
- For industrial waste used in above-ground construction facilities in urban areas or for development in the local plan, f_1_ = 2.0 and f_2_ = 400 Bq/kg.
- For industrial waste used in above-ground parts of construction facilities outside of urban areas or for levelling undeveloped areas, f_1_ = 3.5 and f_2_ = 1000 Bq/kg.
- For industrial waste used in underground construction facilities and structures, including tunnels, but not mine workings, f_1_ = 7.0 and f_2_ = 2000 Bq/kg.

When industrial waste is used for levelling or construction of roads, sports and recreational facilities by the above values, the dose rate absorbed at a height of 1.00 m must be reduced to a value not exceeding 0.3 micrograms per hour (µGy/h) above the surface of the land, road, or object by adding layer of alternate material [35].

The formula for determining the radioactive concentration index of radioactive isotopes of potassium K-40, radium Ra-226 and thorium Th-232 (index I) is given by Formula (1):

(1) $$I=\frac{C_{K-40}}{3000[Bq/kg]}+\frac{C_{Ra-226}}{300[Bq/kg]}+\frac{C_{Th-232}}{200[Bq/kg]}\leq \;1$$

where:

*C_K_*-40, *C_Ra_*-226 and *C_Th_*-232—mean the radioactive concentrations of potassium isotopes *K*-40, radium *R_a_*-226 and thorium *T_h_*-232, respectively, expressed in becquerels per kilogram (Bq/kg).

The results of the natural radioactivity test presented in Table 4 and Table 5 prove that the slag meets the requirements mentioned above. A higher specific weight characterizes concrete produced based on ISF slag than concrete containing only sand. This makes it suitable for producing acoustic screens and heavy concrete. Concretes containing slag have higher gamma absorption than regular concrete. The results [38,39,40] proved that ISF slag can improve the durability and radiological properties of mortar and concrete mixes even for higher replacements and durability properties of SCC mixes for replacements up to 25%. 

### 2.2. Materials and Methodology Preparation of Cement Mortar 

In Table 6, the chemical composition of CSA cement is shown. CSA cement is a mineral hydraulic binder known for its low shrinkage, early strength, and sulphate resistance. The primary components of CSA cement are calcium sulfate aluminate anhydride (4CaO·3Al_2_O_3_·SO_4_), which is responsible for the early increase in strength, dicalcium silicate (delete) (2CaO·SiO_2_), which provides significant strength to the concrete after 28 days, and gISF (CaSO_4_·2H_2_O). CSA cements are fired at a temperature of 1250 °C, which significantly reduces energy consumption during production. The obtained CSA clinker is softer than Portland cement clinker and requires less grinding energy. Ettringite formation takes place during the hardening process according to simplified Formula (2):

(4CaO·3Al_2_O_3_·SO_4_) + 8(CaSO_4_·2H_2_O) + 6Ca(OH)_2_ + 74H_2_O = 3(3CaO·Al_2_O_3_·3CaSO_4_·32H_2_O) (2)

The reaction between calcium sulphate aluminate (4CaO·3Al_2_O_3_·SO_4_) and water can cause expansion of the initial raw materials. This reaction produces ettringite (3CaO·Al_2_O_3_·3CaSO_4_·32H_2_O), which can cause a final expansion of the volume of the initial raw materials. However, if sulphate saturation is low, the reaction produces calcium aluminate monosulfate (3CaO·Al_2_O_3_·CaSO_4_·12H_2_O) with much smaller expansion. The structures of both compounds are similar, making it difficult to distinguish them. Research has been conducted with different proportions in the triangle of materials: calcium sulphate aluminate, gISF (CaSO_4_·2H_2_O), and dicalcium silicate (2CaO·SiO_2_), to determine the proportions of mixtures for fast-hardening, expansive and weakly expansive cement. The CSA cement series includes various products that can be used as a primary binder or accelerator for Portland Cement and are suitable for multiple applications. When used as the primary binder, they can set from a few minutes to hours, gain strength rapidly, and achieve compressive strengths ranging from 50 to 100 MPa [41].

Cement mortars (Table 7) were prepared according to EN 196-1:2016-07 [42]. EN 196-1 states that mortar preparation involves following a specific methodology to meet the required standards and specifications. After one day of hardening, the specimens of cement mortars were carried in water for 27 days.

### 2.3. Materials and Methodology Preparation of a Geopolymer Mortar 

In the late 1970s, Joseph Davidovits, the inventor and developer of a geopolymerisation, coined the term “a geopolymer” to classify the newly discovered synthesis that produces inorganic geopolymeric materials now used for several industrial applications. He also set a logical scientific terminology based on different chemical units, essentially for silicate and aluminosilicate materials, classified according to the Si:Al atomic ratio:

- Si:Al = 0, siloxo,
- Si:Al = 1, sialate (acronym for silicon-oxo-aluminate of Na, K, Ca, Li),
- Si:Al = 2, sialate-siloxo,
- Si:Al = 3, sialate-disiloxo,
- Si:Al > 3, sialate link.

This terminology was presented to the scientific community at an IUPAC conference in 1976. For details, see the Library Milestone Paper IUPAC-76 [43].

The analysed research used metakaolin to create a geopolymer mortar binder, as described in Table 8. According to [43], it is sialate, a geopolymer (-Si-O-Al-O-sialate, poly(sialate)).

An appropriate alkaline activator must be chosen to activate aluminosilicate materials with a low amount of calcium compounds in their composition. Previous research on copper slag alkaline activation has shown that the reaction between ground copper slag and the alkaline activator (either NaOH or sodium water glass) results in the formation of low-base hydro silicates of the calcium silicate hydrate (C-S-H) type, hydrated low-base aluminate and aluminosilicate of the hydro garnet type, calcite, magnesium hydro silicates, mixed sodium-potassium compounds, and alkaline hydrated aluminosilicates of the hydro nepheline, analcime, and natrolite type. These resulting products in the form of hydrates differ significantly from those from the hydration of traditional common-use cement rich in CaO. As the CaO content decreases, the content of the C-S-H and calcium aluminate hydrate (C-A-H) phases formed because of hydration decreases and the zeolite-like phases increase. Choosing the appropriate type and amount of activator is complex and depends mainly on the slag’s chemical composition and specific surface area. The dissolution of aluminium and silica is faster, and the higher the system’s pH is, the more it depends on the quality and content of the activator. Therefore, a higher molar concentration of the alkaline solution with the scale given in Table 9 was used. 

Geopolymer samples (Table 9) were prepared as follows: first, an alkaline solution cooled to 20 degrees Celsius was mixed for 5 min using a sonicator, and then it was added to the previously mixed ISF slag with metakaolin. Everything was mixed in an automatic mortar mixer for cement mortar acc. to EN 196-1:2016-07 [42]. The geopolymer samples were activated at a temperature of 80 °C for 72 h and tightly wrapped in foil to protect against drying and shrinkage. Geopolymer mortars, after thermal activation, were matured in air-dry conditions.

### 2.4. Methodology of Research of Fresh and Hardened Mortar 

The consistency of the cement and a geopolymer mortar was determined per EN 1015-3 [44]. 

Mechanical properties of mortars were investigated acc. to EN 196-1:2016-07 [42], and porosity tests were conducted on three cement and three geopolymer samples. 

The porosity tests of geopolymer and cement mortar were conducted using the following test equipment:

- helium density—AccuPyc II 1340 device from Micromeritics Instruments (Norcross, GA, USA),
- pore size distribution—Poremaster 60 by Quantachrome Instruments (Boynton Beach, FL, USA).

Finally, the immobilisation efficacy of ISF slag in a geopolymer and cement binders was assessed through leachability tests based on the EN 12457-2 standard [45]. An aqueous extract was prepared from the collected waste at a liquid-to-solid phase ratio of L/S = 10 L/kg and then subjected to leaching tests using demineralized water. The sample was shaken for 24 h, allowed to settle for 15 min, and filtered through a filter [45]. The extract was then subjected to several determinations, including pH [46], chloride content [47], sulphate content [48], and the content of sodium, calcium, potassium, lithium, barium [49], phosphorus [50], and fluorides [51]. The AVANTA PM atomic absorption spectrometer from GBC evaluated the heavy metal content in water extracts. The results were compared with the maximum limit values specified in the Regulation on substances particularly harmful to the aquatic environment and conditions to be met when discharging sewage into waters or the ground, as well as when discharging rainwater or snowmelt into water or water facilities (Journal of Laws 2019, item 1311) [34]. This study’s results will be valuable to industries that produce ISF slag and researchers and policymakers working on environmental protection and the responsible handling of industrial waste.

## 3. The Results and Discussion

### 3.1. Test Results of Fresh Mortars Testing

**Figure 4:**
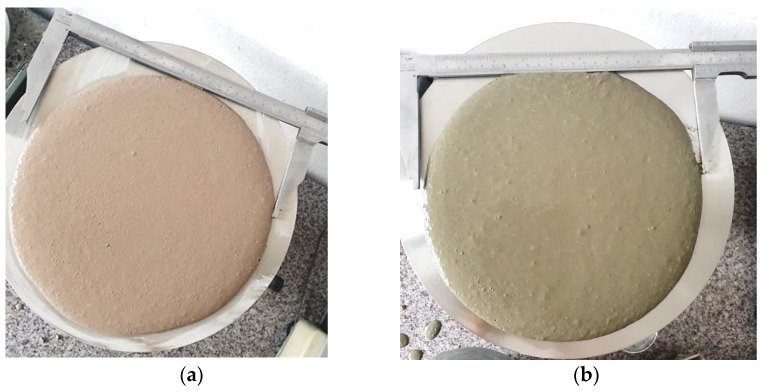
View of the mortar flow diameter, (**a**) a geopolymer, (**b**) cement.

The results for mortar consistency are presented in Table 10 and Figure 4 respectively. As proved, the ISF slag, without 0.125 mm in diameter grains, allows the achievement of high-flow diameter mortars. Moreover, a geopolymer mortar was more stable than cement mortar because geopolymers’ plastic viscosity and adhesion force are very high. Due to its high cohesion, the geopolymer in its liquid state is less susceptible to segregation than cement mortar. On the other hand, this feature does not facilitate increasing the fluidity of the geopolymer; therefore, instead of increasing the share of alkaline solution, it is necessary, as the authors did, to use superplasticizing admixtures resistant to high alkaline pH, preferably other than naphthalene, due to the research results published in the publication [52].

### 3.2. The Results of Hardened Mortar Testing

**Figure 5:**
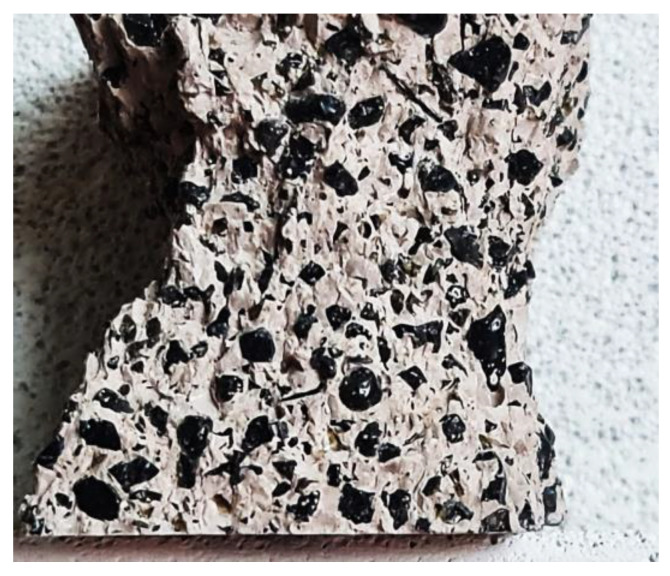
View of the geopolymer mortar breakthrough.

According to the authors’ research findings, they use ISF slag with grain sizes between 0.125 mm and 2 mm, resulting in high-strength self-compacting geopolymer and cement mortars. The results presented in Table 11 and Figure 5 demonstrate that IPS slag is an excellent alternative to natural sand. For instance, 100% substitution of sand with slag resulted in a compressive strength of about 110 MPa for the CSA mortar and over 140 MPa for the geopolymer. Such compressive strength corresponds to ultra-high-strength mortars, thanks to the properties of the slag [19], strong adhesion between the cement pastes and slag grain, and appropriate consistency of the mortars (Table 10). 

An important finding is the significantly higher tensile strength of the geopolymer, which exceeds that of cement mortar by a factor of two (as shown in Table 11). As a polymer matrix, the geopolymer exhibits an exceptional capacity to withstand tensile forces, which reduces the risk of surface scratches. It has excellent mechanical properties and better adhesion to the cement slurry than natural aggregate. Due to the rough texture of the ISF particles, the ITZ formed between the ISF slag and cement particles is denser, thus reducing water absorption by 20–25% in mixtures with 60% ISF slag. The flexural and peel strengths of most concrete containing ISF slag amounts up to 70% were the same or higher than those of the control mixtures. These factors contributed to low air content in the mortars, which is responsible for the high strength. Previous works [38,53] have shown that slag from a zinc smelter is characterised by favourable grain composition, good compatibility, and high strength parameters such as internal friction angle and cohesion.

**Figure 6:**
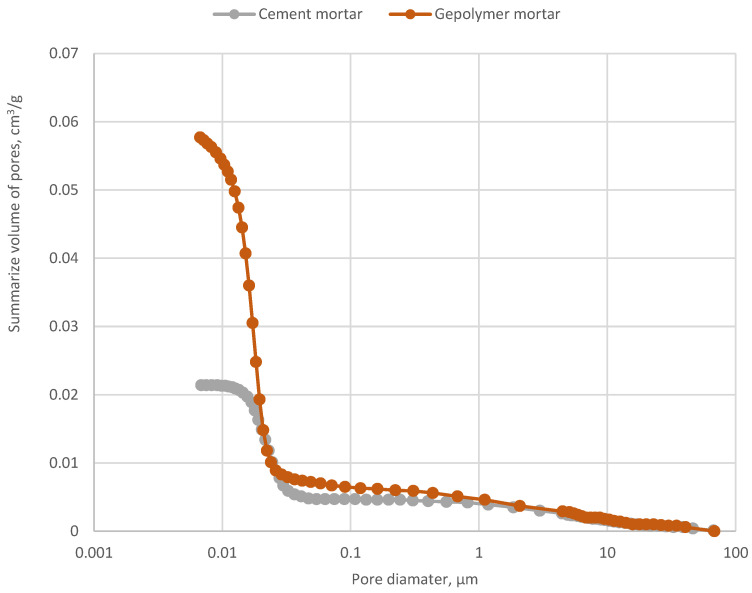
Comparison of pore size distribution (μm) for a geopolymer and cement samples (g/cm^3^) from ISF slag.

The above conclusions also confirm the porosity characteristics of investigated mortars. Research results from three test samples in Figure 6 demonstrate that the geopolymer has a denser structure. The porosity calculations, based on helium density measurements, are as follows:

- cement mortar—6.13%,
- a geopolymer mortar—12.48%.

The results of determining the helium density (dHe) values (mean value of 20 measurements and standard deviation) confirm the correlation between porosity and mechanical parameters of mortar:

- cement mortar—3.0527 g/cm^3^ ± 0.0020 g/cm^3^,
- a geopolymer mortar—2.4706 g/cm^3^ ± 0.0020 g/cm^3^.

### 3.3. Comparison of the Leachability of Slag and Geopolymers and Cement Mortars—Assessment of the Environmental Impact of Mortars

The SEM analysis of the microstructure of the cement paste showed that the interfacial transition zone (ITZ) between the ISF particles and the cement paste was denser and more compact than the ITZ between the cement-sand paste with good bond strength. FESEM-EDS analysis performed on alkali-activated materials confirmed the physical confinement of heavy metals: Zn, Cr, Pb and Cu [3,53].

High density and low porosity of the microstructure of mortar or concrete are essential, and not the most important, conditions for achieving low leachability of heavy metals.

The publications [17,21,22,23,24] suggested the possibility of a geopolymerisation of ISF slag. The study is part of our activities towards the complete utilisation of slag for building material applications using a geopolymerization process, which involves the formation of a new rock-like species from various aluminosilicate minerals under a strongly alkaline environment. Additionally, the geopolymer prepared from ISF slag demonstrated higher compressive strength values than those prepared from other types of slag, indicating its enhanced reactivity. The prepared samples were subjected to TCLP tests, which confirmed that the release of toxic metals was within the limits set by the USEPA. Therefore, this process is considered environmentally friendly when utilised for zinc slag.

That is very important, as the cement binder and a geopolymer binder characterise different pH values: CSA pH = 11 and a geopolymer pH = 14, critical parameters for heavy metals’ leachability results. The pH value of a solution significantly influences the leachability of heavy metals from cement-based building materials. When the pH of a solution is acidic, heavy metals are more likely to be leached out of the cement matrix and into the surrounding environment. This is because the acidic conditions can dissolve the cement matrix and release the heavy metals trapped within it. On the other hand, when the pH of a solution is alkaline, the leachability of heavy metals from cement-based building materials is reduced. Alkaline conditions can help stabilise the heavy metals within the cement matrix, preventing them from leaching out into the environment. Overall, a solution’s pH value significantly impacts the leachability of heavy metals from cement-based building materials. It is essential to consider the pH value of the surrounding environment when designing and constructing cement-based structures to minimise the leaching of heavy metals. 

We proved that the geopopolrysation of ISF slag is not an environmentally friendly solution. Table 12 summarises the results of tests conducted on the ability of cement and geopolymer materials to contain ISF waste. The geopolymer mortar exceeded the barium content limit. As a result, it is recommended to use cement mortar with CSA cement, which has a lower pH and a higher portlandite content than CEM. This solution is more effective at immobilising ISF waste. The leachability of barium increases with the pH of the matrix. When testing the leachability of IPS slag with water (as shown in Table 9), the leachability of this compound is found to be low. However, the leachability increases significantly in the case of a geopolymer mortar with a solution pH of 14 (as indicated in Table 12). Barium leachability is within acceptable limits at pH 12 (cement mortar with CSA).

Based on the analysis of the results, it has been found that a cement matrix is a better option than a geopolymer mortar for immobilising ISF waste due to the leaching of barium. However, the geopolymer mortar has a very alkaline reaction to the washed liquid and does not meet environmental guidelines. Considering the results obtained, further research should be focused on using ISF slag as a substitute for sand or fine aggregate and incorporating it into traditional cement mortars and concrete rather than geopolymer ones. 

## 4. Conclusions

In the scope of used materials and obtained results, the following conclusions were achieved:

- ISF slag meets the requirements for leachability of hazardous compounds and radioactivity. We proved that. geopolymerisation is not an adequate way to create an environmentally friendly solution. Due to the leachability of barium, the cement matrix is a better option than a geopolymer mortar for immobilising ISF waste. The results of porosity characteristics demonstrated that the geopolymer has a denser structure.
- The results of the natural radioactivity test proved that the ISF slag meets the requirements for building materials.
- The use of CSA cement helps reduce the time needed for mortar. The flowability of self-compacting cement or a geopolymer mortar with 100% replacement sand with ISF slag is very favourable, which allows for the elimination of mechanical compaction of payment to achieve low porosity and a very dense microstructure.
- The research results have shown that by removing grains smaller than 0.125 mm and more significant than 2 mm, ISF slag can produce high-strength self-compacting geopolymer and cement mortars. Geopolymers have better mechanical properties, particularly tensile strength, and are twice as big as cement specimens. This mechanical parameter is caused by the stronger adhesion of slag grains to the binder and the higher bending strength of a geopolymer binder, just like polymer concrete. A geopolymer mortar achieved up to 140 MPa, while cement mortar achieved an average of 100 MPa.

**Figure 7:**
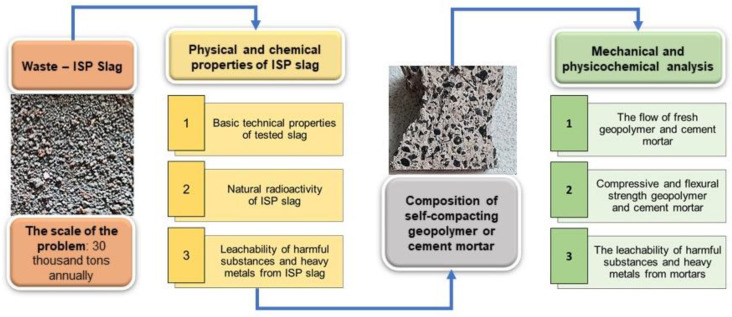
Authors’ new way to achieve high: flowable, quick setting, mechanical strength, and environmentally friendly mortars or concrete with ISF slag.

The authors graphically summarized the obtained conclusion in Figure 7.

Finally, it should be noted that CSA cement should not be used in reinforced concrete. Unfortunately, the lime in CSA is firmly bound, resulting in alkalinity ranging from 10.5 to 11 pH; it is not adequate for the passivity of reinforced concrete and is exposed to carbonation. The publication’s authors [16] found that reinforcement corrosion was not expected in concrete containing Portland cement and 100% replacement sand with ISF slag. They also observed that ettringite, a mineral that strengthens the concrete, was more frequently found in elements subjected to extraction in concrete with 100% content ISF slag. This mineral helped to tighten larger pores in the concrete but could cause the filling out of tiny pores in the longer process. However, the authors also noticed that the concentration of chloride ions in concrete significantly slowed the corrosion processes, particularly in the reference concrete and concrete containing up to 25% ISF slag.

Due to the concrete’s shrinkage, at least 10% of the CSA cement content should be supplemented with Portland cement. This will not significantly affect the setting time and tightness [54,55,56].

## Figures and Tables

**Table 1:** Chemical composition of ISF slag.

No.	Component	Amount, [%]
1.	SiO_2_	24.4
2.	Al_2_O_3_	15.3
3.	CaO	17.6
4.	MgO	6.4
5.	Fe_2_O_3_	27.9
6.	Zn	5.8
7.	Pb	4.7 ± 1.3
8.	Weight gain from 430 to 1000 °C	4.2%

**Table 2:** Results of pH slag ISF measurements.

No.	Component	Value of pH		T [°C]
		pH Meter	Test Paper	
1.	ISF slag	9.7	9.0	19.9

**Table 3:** The results of the tests on the leachability of harmful substances and heavy metals from slag ISF are expressed in mg/L.

Parameter	Symbol	ISF Slag	Highest Permissible Value [34]
pH	pH	7.9	6.0–9.0
Hexavalent chromium	Cr^6+^	blq *	0.1
Chromates	Cr^3+^	blq *	0.1
Cobalt	Co	blq *	1
Lead	Pb	0.28	0.5
Nickel	Ni	0.27	0.5
Zinc	Zn	0.27	2
Copper	Cu	0.07	0.5
Cadmium	Cd	0.02	nr **
Sulphate	SO_4_^2−^	68.91	500
Chloride	Cl^−^	69.12	1000
Fluoride	F	0.29	25
Phosphorus	P	0.07	2
Potassium	K	3.72	80
Sodium	Na	23.8	800
Barium	Ba	0.39	2
Iron	Fe	0.14	10
Manganese	Mn	0.005	nr **

* blq—values below the limit of quantification, ** nr—no requirements.

**Table 4:** Natural radioactivity of ISF slag.

Material	Radioisotope Concentrations [Bq/kg]			Radioactive Concentration Index of Radioisotopes
	*C_K_*_-40_	*C_Ra_*_-226_	*C_Th_*_-232_	*I* [-]
ISF slag	266 ± 51	69 ± 12	31 ± 5	0.47 ± 0.08

**Table 5:** Natural radioactivity slag ISF.

Material	Maximum Radionuclide Concentrations [Bq/kg]			Activity Indicators	
Parameter	*K*-40	*Ra*-226	*Th*-232	f_1_ max [-]	f_2_ max [Bq/kg]
ISF slag	225.81	147.99	27.29	0.705	147.99

**Table 6:** Chemical composition of CSA cement.

Component	Contents %
CaO	45
SiO	8
Al_2_O_3_	20
Fe_2_O_3_	3
SO_3_	20
MgO	2.5

**Table 7:** Composition of self-compacting cement mortar.

Component	Unit	Cement Mortar
CSA, sulphide alumina cement, 52.5R	grams	450.0
Water	grams	193.50
ISF slag	grams	675.00
Water or alkali/binder	-	0.43
Powdered naphthalene fluidising admixture	grams	9.0

**Table 8:** Chemical composition of metakaolin.

Component	SiO_2_	TiO_2_	Al_2_O_3_	Fe_2_O_3_	MgO	CaO	Na_2_O	K_2_O	P_2_O_3_	SO_3_	Σ
[% by weight.]	53.3	0.5	42.5	0.4	0.1	0.1	2.0	0.6	0.2	0.1	99.8

**Table 9:** Composition of self-compacting geopolymer or cement mortar.

Component	Unit	A Geopolymer Mortar
Metakaolin	grams	450.00
Water	grams	60.33
NaOH (M6.9)	grams	16.65
Soda water glass, solid parts 47%	grams	130.51
ISF slag	grams	670.00
Powdered naphthalene superplasticizing admixture	grams	9.0

**Table 10:** The flow of self-compacting cement and a geopolymer mortar.

Type of Mortar	Flow Diameter, cm
Cement mortar	28
A geopolymer mortar	27

**Table 11:** Compressive and flexural strength results of 28 days of mortar.

Type of Mortar	28 Days Compressive Strength, MPa	28 Days Tensile Strength, MPa
Cement mortar with ISF slag	113.1	12.4
	110.4	13.1
	110.0	12.6
	111.3	
	114.6	
	112.6	
A geopolymer mortar with ISF slag	139.9	31.4
	140.2	32.2
	142.4	32.6
	143.5	

**Table 12:** Results of leachability of harmful substances and heavy metals from mortars, expressed in mg/L, except pH.

Parameter	Symbol	Geopolymer Mortar	Cement Mortar	Highest Permissible Value [34]
pH	pH	10.7	9.5	6.0–9.0
Chloride	Cl^−^	69.12	69.12	1000
Sulphate	SO_4_^2−^	171.55	200.76	500
Phosphorus	P	0.16	0.07	2
Potassium	K	1.50	6.79	80
Sodium	Na	186.00	9.82	800
Barium	Ba	29.20	0.65	2
Zinc	Zn	0.06	blq *	2
Copper	Cu	blq *	blq *	0.5
Lead	Pb	0.06	blq *	0.5
Cadmium	Cd	0.001	0.004	nr **
Chrome	Cr	0.07	0.07	0.1
Cobalt	Co	blq *	blq *	1
Iron	Fe	blq *	blq *	10
Manganese	Mn	0.007	0.005	nr **
Nickel	Ni	0.30	0.37	0.5

* blq—values below the limit of quantification, ** nr—no requirements.

## Data Availability

The raw data supporting the conclusions of this article will be made available by the authors on request.

## Abbreviations

ISF	imperial smelting furnace
CSA	calcium sulfoaluminates cement
SCC	self-compacting concrete

## References

[1] Kozioł W., Kawalec P. (2008). Alternative aggregates in construction. Mod. Eng. Constr..

[2] National Waste Management Plan 2014, Annex to Resolution No. 217 of the Council of Ministers of 24 December 2010, Warsaw 2010. https://isap.sejm.gov.pl/isap.nsf/DocDetails.xsp?id=wmp20101011183.

[3] Road and Bridge Research Institute in Warsaw (2004). Assessment and Testing of Selected Industrial Waste for Use in Road Structures.

[4] Statistical Yearbook of Industry Central Statistical Office, Warsaw, 2014. https://stat.gov.pl/obszary-tematyczne/roczniki-statystyczne/roczniki-statystyczne/rocznik-statystyczny-przemyslu-2014,5,8.html.

[5] (2011). Strategies and technological scenarios for developing and using rock raw material deposits. Vol. 1.3.3 Assessment of the Possibility of Substituting Rock Raw Materials to Meet Demand.

[6] Annual Review: European Aggregates Association. Brussels, 2013. https://www.aggregates-europe.eu/publications/.

[7] Municipal Waste Management in Poland Analysis of waste management costs—Assessment of investment needs in the country in the field of waste prevention and waste management in connection with the new EU financial perspective 2021–2027, Warsaw 2020. https://ios.edu.pl/strona-glowna/analiza-kosztow-gospodarki-odpadami-komunalnymi/.

[8] Regulation EU 2020/852 of the European Parliament and of the Council of 18 June 2020 on the Establishment of a Framework to Facilitate Sustainable Investment, and Amending Regulation (EU) 2019/2088 (Text with EEA Relevance) PE/20/2020/INIT. http://data.europa.eu/eli/reg/2020/852/oj.

[9] Babu V., Kabeer K.S.A., Vyas A.K. (2022). A review of the characterization and use of ISF slag as fine aggregate in cement concrete. Mater. Today Proc..

[10] Ferreira V.J., Vilaplana A.S.D.G., García-Armingol T., Aranda-Usón A., Lausín-González C., López-Sabirón A.M., Ferreira G. (2016). Evaluation of the steel slag incorporation as coarse aggregate for road construction: Technical requirements and environmental impact assessment. J. Clean. Prod..

[11] Atzeni C., Massidda L., Sanna U. (1996). Use of Granulated Slag from Lead and Zinc Processing in Concrete Technology. Cem. Concr. Res..

[12] Morrison C., Hooper R., Lardner K. (2003). Ferro-silicate slag from ISF zinc production is used as a sand replacement in concrete. Cem. Concr. Res..

[13] Weeks C., Hand R.J., Sharp J.H. (2008). Retardation of cement hydration caused by heavy metals present in ISF slag used as an aggregate. Cem. Concr. Compos..

[14] Szweda Z., Mazurkiewicz J., Konečný P., Ponikiewski T. (2023). Effect of Imperial Smelting Process Slag Addition in Self Compacting Concrete Concrete on the Efficiency of Electrochemical Chloride Extraction. Materials.

[15] Pozzi M., Nowińska K. (2006). Distribution of selected elements accompanying the ISF technological process in HC Miasteczko Śląskie in terms of their recovery and impact on the environment. Econ. Miner. Resour..

[16] Szweda Z., Ponikiewski T., Katzer J. (2017). A study on replacement of sand by granulated ISF slag in SCC as a factor formatting its durability against chloride ions. J. Clean. Prod..

[17] Alex T.C., Kalinkin A.M., Nath S.K., Gurevich B.I., Kalinkina E.V., Tyukavkina V.V., Kumar S. (2013). Utilization of zinc slag through a geopolymerization: Influence of milling atmosphere. Int. J. Miner. Process..

[18] Pozzi M., Nowińska K. (2010). Estimation of the amount of heavy metals in the hazardous waste landfill of Zinc Smelter “Miasteczko Śląskie”. Min. Geol..

[19] Kanneboina Y.Y., Kabeer K.S.A., Bisht K. (2023). Valorization of lead and zinc slags for the production of construction materials—A review for future research direction. Constr. Build. Mater..

[20] Alwaeli M. (2013). Application of granulated lead—Zinc slag in concrete as an opportunity to save natural resources. Radiat. Phys. Chem..

[21] Xia M., Muhammad F., Zeng L., Li S., Huang X., Jiao B., Shiau Y., Li D. (2019). Solidification/stabilization of lead-zinc smelting slag in composite based a geopolymer. J. Clean. Prod..

[22] Zhang P., Muhammad F., Yu L., Xia M., Lin H., Huang X., Jiao B., Shiau Y., Li D. (2020). Self-cementation solidification of heavy metals in lead-zinc smelting slag through alkali-activated materials. Constr. Build. Mater..

[23] Chen W., Peng R., Straub C., Yuan B. (2020). Promoting the performance of one-part alkali-activated slag using fine lead-zinc mine tailings. Constr. Build. Mater..

[24] Prasad P.S., Ramana V.G. (2016). Imperial smelting furnace (zinc) slag as a structural fill in reinforced soil structures Geotext. Geotext. Geomembr..

[25] Saedi A., Jamshidi-Zanjani A., Khodadadi A., Mohseni M., Nejati H. (2022). Utilization of lead—Zinc mine tailings as cement substitutes in concrete construction: Effect of sulfide content. J. Build. Eng..

[26] Pozzi M., Nowińska K. (2006). The content of accompanying elements in the materials of the technological process of ISF Huta Cynku “Miasteczko Śląskie”. Miner. Resour. Manag..

[27] de Andrade Lima L.R.P., Bernardez L.A. (2011). Characterisation of the lead smelter slag in Santo Amaro, Bahia, Brazil. J. Hazard. Mater..

[28] Chen D.T., Roy A., Li Y.Q., Bogush A., Au W.Y., Stegemann J.A. (2023). Speciation of toxic pollutants in Pb/Zn smelter slags by X-ray Absorption Spectroscopy in the context of the literature. J. Hazard. Mater..

[29] (2003). Instruction № 234/2003, Testing of Natural Radioactivity of Raw Materials and Building Materials.

[30] IBDiM Technical Approval № AT99-04-0538: ISF Slag Granules; Zinc Smelter “Miasteczko Śląskie”: Miasteczko Śląskie, Poland, 2011. https://www.ibdim.edu.pl/pl/oferta/european-technical-assessment-eta.

[31] Alwaeli M. (2017). Investigation of gamma radiation shielding and compressive strength properties of concrete containing scale and granulated lead-zinc slag wastes. J. Clean. Prod..

[32] Tripathi B., Chaudhary S. (2016). Performance-based evaluation of ISF slag as a substitute for natural sand in concrete. J. Clean. Prod..

[33] A Geopolymer Institute. https://www.geopolymer.org/science/introduction/.

[34] Regulation on Substances Particularly Harmful to the Aquatic Environment and Conditions to Be Met When Discharging Sewage into Waters or into the Ground, as Well as When Discharging Rainwater or Snowmelt into Water or into Water Facilities (Dz.U. 2019, poz. 1311). https://isap.sejm.gov.pl/isap.nsf/DocDetails.xsp?id=WDU20190001311.

[35] Yin N.H., Sivry Y., Guyot F., Lens P.N., van Hullebusch E.D. (2016). Evaluation of chemical stability of lead blast furnace (LBF) and imperial smelting furnace (ISF) slags. J. Environ. Manag..

[36] (2012). Tests of Geometric Properties of Aggregates. Part 1: Determination of Grain Composition. Sieving Method.

[37] Regulation of the Council of Ministers of 2 January 2007. on the Requirements for the Content of Natural Radioactive Isotopes of Potassium K-40, Radium Ra-226 and Thorium Th-228 in Raw Materials and Materials Used in Buildings Intended for the Stay of People and Livestock, as Well as in Industrial Waste Used in Construction, and Control of the Content of These Isotopes (Dz.U. 4, poz. 29). https://isap.sejm.gov.pl/isap.nsf/DocDetails.xsp?id=WDU20070040029.

[38] European Commission Radiation Protection 112. Radiological Protection Principles Concerning the Natural Radioactivity of Building Materials. Directorate—General; Environment, Nuclear Safety and Civil Protection, 1999. https://www.scirp.org/reference/ReferencesPapers?ReferenceID=1077862.

[39] Regulation of the Council of Ministers of 17 December 2020. on Building Materials for Which the Radioactive Concentration of Radioactive Isotopes of Potassium K-40, Radium Ra-226 and Thorium Th-232 is Determined, the Requirements for Making These Determinations and the Value of the Radioactive Concentration Indicator, the Exceedance of Which Is Reported to the Competent Authorities (Journal of Laws Laws of 7 January 2021, Item 33). https://isap.sejm.gov.pl/isap.nsf/DocDetails.xsp?id=WDU20210000033.

[40] Zawisza E. (2007). Compactability and shear strength of slag from a zinc smelter. Scientific works of the Institute of Geotechnics and Hydrotechnics of the Wrocław University of Science and Technology. Conferences.

[41] Polar Bear CSA Cement Product Information. www.csa-cement.com.

[42] (2016). Cement Testing Methods—Part 1: Determination of Strength.

[43] Davidovits J. Solid Phase Synthesis of a Mineral Blockpolymer by Low Temperature Polycondensation of Aluminosilicate Polymers, IUPAC International Symposium on Macromolecules Stockholm; Sept. 1976; Topic III, New Polymers of high stability. http://www.geopolymer.org/library/technical-papers/20-milestone-paper-iupac-76/.

[44] (2000). Test Methods for Mortars for Walls—Determination of the Consistency of Fresh Mortar (Using a Flow Table).

[45] (2006). Characterization of Waste. Compliance Test for Leaching of Granular Waste Materials and Sludge. Part 2: One-Stage Batch Test with a Liquid/Solid Ratio of 10 l/kg for Materials with a Particle Size of Less Than 4 mm (No or with a Size Reduction).

[46] (2012). Determination of pH.

[47] (1994). Determination of Chlorides. Titration Method with Silver Nitrate in the Presence of Chromate as an Indicator (Mohr Method).

[48] (2002). Determination of Sulphates (VI)—Gravimetric Method with Barium Chloride.

[49] (1994). Determination of Sodium and Potassium by Flame Emission Spectrometry.

[50] (2006). Determination of Phosphorus. Ammonium Molybdate Spectrometric Method.

[51] REF 918142, Test 1-42, Standard Methods for the Examination of Water and Wastewater (4500-F–D), Fluoride. https://sitefiles.camlab.co.uk/918142en%20(1).pdf.

[52] Jia C., Batterman S. (2010). A Critical Review of Naphthalene Sources and Exposures Relevant to Indoor and Outdoor Air. Int. J. Environ. Res. Public Health.

[53] Gruchot A., Michalski P., Zawisza E., Sarsby R.W., Felton A.J. (2007). Shearing strength of coal mining wastes used as coarse-grained building soils. W: Geotechnical and Environmental Aspects of DISFosal Sites, Proceedings of the 4th International Symposium on Geotechnics Related to the Environment—Green 4, Wolverhampton, UK, 28 June–1 July 2004.

[54] Kurdowski W. (2010). Chemistry of Cement and Concrete. Cement Producers Association.

[55] Lee K.H., Nam S.Y., Min S.E., Lee H.W., Han C., Ahn J.W. (2016). Characterizations of high early-strength type shrinkage reducing cement and calcium sulfo-aluminate by using industrial wastes. J. Korean Ceram. Soc..

[56] Maurcio M., Costa U. Influence of the calciumsulphate and w/c-ratio on the hydration of calciumsulfoaluminate cement. Proceedings of the Conference Materials from the 13th International Congress of Cement Chemistry.

